# Impact of group antenatal care on lactational amenorrhea method awareness and knowledge: A cluster randomized control trial

**DOI:** 10.1371/journal.pone.0333074

**Published:** 2025-10-15

**Authors:** Theresa Norpeli Lanyo, John E. O. Williams, Bidisha Ghosh, Veronica E. A. Apetorgbor, Vida A. Kukula, Ruth Zielinski, Elizabeth Awini, Cheryl A. Moyer, Jody Lori

**Affiliations:** 1 Department of Health Behavior and Biological Sciences, University of Michigan School of NursingAnn Arbor, Michigan, United States of America; 2 Dodowa Research Center, Ghana Health Service, Greater Accra, Ghana; 3 Applied Biostatistics Laboratory, University of Michigan School of Nursing, Ann Arbor, Michigan, United States of America; 4 Dodowa Research Center, Ghana Health Service, Greater Accra, Ghana; 5 Department of Health Behavior and Biological Sciences, University of Michigan School of NursingAnn Arbor, Michigan, United States of America; 6 Department of Learning Health Sciences, University of Michigan Medical School, Ann Arbor, Michigan, United States of America; 7 Department of Health Behavior and Biological Sciences, University of Michigan School of Nursing, Ann Arbor, Michigan, United States of America; College of Medicine, University of Ibadan, NIGERIA

## Abstract

High fertility rates in low and middle-income countries (LMICs), especially in sub-Saharan Africa and Ghana, lead to closely spaced pregnancies and contribute to high maternal and infant morbidity and mortality. Family planning remains critical for reducing rapid repeat and unwanted pregnancies, thus improving the health and survival outcomes. Unfortunately, many women face significant unmet needs due to limited knowledge, misinformation, and limited access to modern family planning methods in particular. Breastfeeding plays a substantial role, and the lactational amenorrhea method (LAM) offers a practical, natural, readily available, and cost-efficient postpartum option when practiced correctly. However, many mothers lack sufficient awareness of LAM and the conditions necessary for its effectiveness. Antenatal care provides a strategic opportunity for targeted education and counselling on contraceptive choices, empowering women with the knowledge to adopt safe, informed, and sustainable practices. Group antenatal care (G-ANC), recommended by WHO for research in LMICs, offers a comprehensive and participatory platform for health education and behavioral change. This study examined G-ANC and its impact on creating awareness and improving knowledge of the lactational amenorrhea method among mothers. A cluster randomized control trial, registered at ClinicalTrials.gov on 25/07/2019 with RCT number NCT04033003, was conducted in Ghana at 14 health facilities with a total of 1761 participants. The study included pregnant women who were at least 15 years old, able to speak English or one of four local languages, and less than 20 weeks of gestation at enrollment. Women classified as high-risk by the midwife were excluded from participation in the study. Facilities were randomized using a matched pair method. Structured interviews were conducted at baseline and subsequent timepoints. The results found that group antenatal care increases maternal awareness and knowledge of the postpartum lactational amenorrhea method of family planning compared to individualized care. Subgroup analysis revealed that a mother’s level of education and parity strongly predict maternal awareness of the lactational amenorrhea method. These findings support group antenatal care as an effective strategy to improve knowledge on the use of lactational amenorrhea as a family planning method.

## Introduction

High fertility rates influence birth rates, which, in turn, affect maternal and newborn morbidity and mortality [1–5]. To enhance maternal and infant health and survival, it is crucial to promote awareness of family planning options and improve knowledge through improved contraceptive education, particularly during the antenatal period [6]. Reducing unintended pregnancies is essential for decreasing the associated health risks [7]. Family planning plays a vital role in safeguarding the health and survival of women and children [8]. Currently, approximately 800 women and 6,500 babies die daily from preventable causes related to pregnancy and childbirth, predominantly in sub-Saharan Africa (SSA) [5,9]. There are about 1.9 billion women aged 15–49, with approximately 1.1 billion requiring family planning services globally [10]. Among these, postpartum women represent the group with the greatest unmet need for family planning, yet often lack the information necessary to achieve safer birth intervals and manage unplanned pregnancies [10,11]. Many remain unaware of available family planning options [12,13] or face access to and use of modern contraception, particularly in SSA [10].

Exclusive breastfeeding is a significant practice in reducing fertility rates [14–17]. And the lactational amenorrhea method (LAM) relies on exclusive breastfeeding to suppress ovulation in the postpartum period [18], offering temporary contraception for women who have not yet resumed their menstrual cycles [19–22]. LAM is recognized as a century-old method with a 98% effectiveness within the first six months of exclusive breastfeeding [18]. It serves as a natural, accessible, and cost-effective family planning option, especially for resource-constrained settings where access to modern methods is often limited. Knowledge of LAM and proper application can help reduce the incidence of rapid repeat pregnancies [23]. However, studies in SSA indicate a lack of awareness regarding LAM and the conditions necessary for its effectiveness [12,13]. Addressing this gap necessitates comprehensive contraception counseling, including couple counseling and user-friendly access, during antenatal and immediate postpartum periods [24–27].

Antenatal care represents a critical platform for health education, often serving as the first structured point of contact for pregnant women. It provides an opportunity to deliver contraception counseling and equip women with clear, practical, and user-friendly guidance to support safe and informed reproductive decision-making [10,11]. In line with WHO’s recommendation to explore group antenatal care (G-ANC) in low and middle-income countries (LMICs) [28], this study rigorously evaluates the model in Ghana, focusing on maternal and infant health outcomes. By examining the integration of LAM into G-ANC, this research addresses a persistent gap in postpartum family planning education while leveraging the innovative, peer-supported, and participatory nature of G-ANC. This focus on both strengthening contraceptive counseling during antenatal care and building capacity for women to utilize available methods when modern contraception is limited underscores the significance of this study.

### Group antenatal care

GANC is a model that shifts away from traditional individualized care. It integrates the concept of ‘knowing,’ which promotes behavioral change through comprehensive interactive, participatory teaching and learning. This approach fosters peer support, educational opportunities, and a shared environment conducive to sharing breastfeeding experiences, knowledge, and practice [29–34]. The intersection between the knowledge approach of GANC and the understanding needed to optimize the effectiveness of LAM presents an opportunity for exploration. GANC sessions serve as a platform for healthcare providers to educate women about various family planning methods, including LAM. Additionally, the peer support and experience exchange within the groups may influence women’s awareness and positive decision-making regarding postpartum contraceptive options, including the adoption of LAM. The study investigated how GANC influences the improvement of maternal knowledge of LAM by hypothesizing that pregnant women randomized into GANC will exhibit improved knowledge of LAM as a family planning option compared to women in the current individualized care.

## Materials and methods

### Study design and setting

This study was part of a larger cluster randomized control trial on GANC registered at ClinicalTrials.gov on 25/07/2019 (RCT: NCT04033003). The study was conducted in 14 health facilities across four districts in the Eastern Region of Ghana from July 2019 to May 2022. The facilities were cluster randomized using matched pair methods (specifically the nbpM matching package from R software version 1.5.0) [35] to ensure the control and intervention facilities were similar in terms of the number of deliveries and the average gestational age of the women at the time of enrollment for antenatal care (ANC) in each facility. Next, for each pair of facilities, one was randomly assigned to receive the intervention (GANC) while the other was assigned to routine individual antenatal care (IANC), which is the current standard of care. Any threat to the internal validity of recruitment and intervention was dealt with through randomization and the use of different locations of health facilities for the groups. The protocol and other details about the study are reported elsewhere [36].

### Ethical approval

Ethical approval for the study was obtained from the Institutional Review Boards of the University of Michigan (HUM#00161464) and the Ghana Health Service (GHS-ERC: 016/04/19). The standard written informed consent procedure was waived to make up for the limitations in the literacy rates among participants.

### Sample

The sample size was calculated for an intraclass correlation coefficient of 0.01 using the CRTSize package from R software at 80% power, with at least 100 women per facility required to achieve the effect [37]. Our analysis focused on three key outcomes: the change in birth preparedness and complication readiness, the percentage of women receiving postpartum checks, and the percentage of babies receiving postnatal check-ups by day two. These indicators helped to evaluate the effectiveness of our initiatives. About 120 women were recruited per facility to preserve power due to attrition [36]. Recruitment was conducted at antenatal clinics within the study facilities, where trained research assistants (RAs) collaborated with midwives to identify women who met the eligibility criteria. Thus, the midwife (as a medical professional) made the assessment that a woman met the inclusion criteria. Eligible participants were required to be less than 20 weeks pregnant, to speak any of the following languages: Dangme, Ga, Akan, Ewe, or English, and to be over the age of 15. Moreover, individuals classified as high-risk by the midwife were excluded from participation in the study.

**Fig 1:**
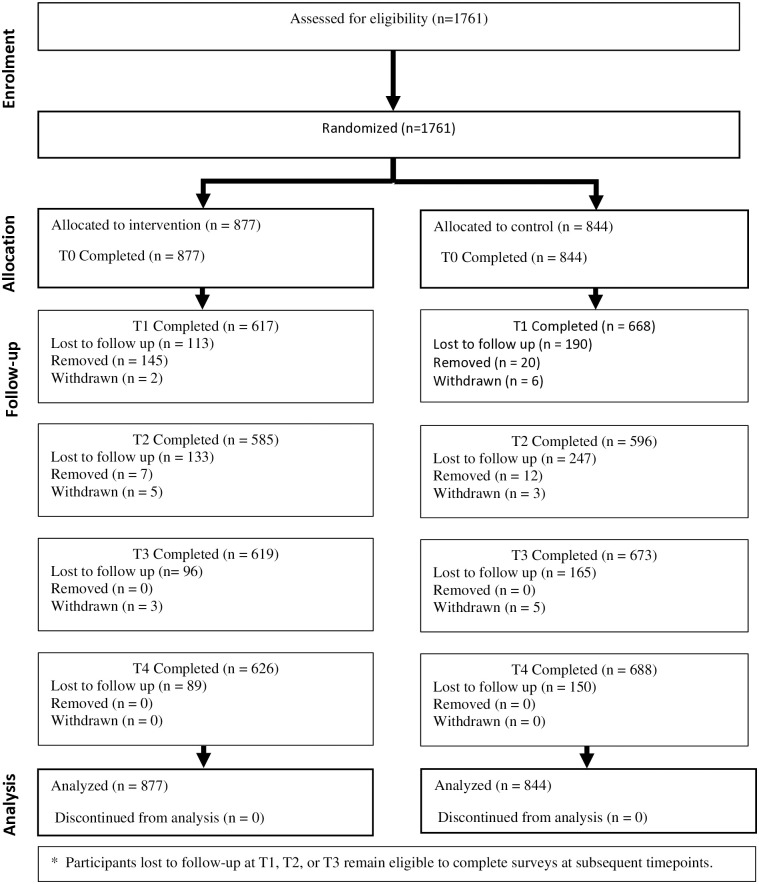
Consort Diagram.

Interested participants were taken through a comprehensive informed consent procedure, including how the results would be used to improve ANC service delivery for better outcomes. Written or verbal informed consent was obtained at recruitment. For those who could not read or write, the consent form (translated into the four major local languages) was read aloud individually to them in private. Participants were allowed to ask questions until the information was clear. Participants signed or marked with a thumbprint, and the consent form was countersigned by the RA obtaining consent. The consort diagram (Fig 1) below depicts the flow of participants through various stages of a trial, including enrollment, intervention allocation, follow-up, and analysis.

### Intervention

The *Maternal Health Literacy Skills Framework* [38], a modified version of the *Health Literacy Skills Framework* by Squiers and colleagues [39], formed the basis for this study. Before the intervention, all research assistants and midwives at both the intervention and control facilities received training on the essential components of ANC based on WHO recommendations to ensure the delivery of quality care at all sites prior to implementation of the intervention. Care providers at the intervention sites were then trained on GANC and delivery methodology for the study, while those in the control site continued with routine individual ANC. The GANC provider training emphasized active listening and the use of picture cards as an important training resource for low-literacy learners. A 3-day didactic training with groups of 10–12 clinical personnel focused on facilitating group ANC, use of the methodology, organizing groups, and an overview of the research. Participants then had the opportunity to practice delivering care using the group model under the support and supervision of the primary investigator and an experienced trainer.

Pregnant women with similar gestational age were placed into groups of 10–14 women. Participants received comprehensive individual assessments with the midwife on their initial visit and eight [8] group sessions following the most recent recommendations from WHO [28]. The sessions involved participants sitting in circles facing each other for roughly 60–90 minutes, engaging in a facilitated discussion led by a midwife. GANC employed context-specific, evidence-based, and tailored health messaging through participatory and educative sessions for maternal, child, and reproductive health. To encourage group discussions, reflections, and interactive learning among participants, strategies such as storytelling, peer support, demonstration, and teach-back, as well as the use of picture cards, were used as tools for these iterative sessions.

### Measures and data collection

Quantitative data were collected at five time points for the larger study: enrollment (T0), 34 weeks of gestation – 3 weeks postpartum (T1), 6–12 weeks postpartum (T2), 6 months postpartum (T3), and one year postpartum (T4). This assessed changes in knowledge and behavior over time using the maternal health literacy (MaHeLi) tool (α = 0.92) [40], the Contraceptive Self-Efficacy among women in sub-Saharan Africa (CSESSA) (α = 0.93) [41], and WHO guideline questionnaires on G-ANC [28]. At T0, demographic information was gathered. Questions from the measures pertaining to having heard about breastfeeding to prevent pregnancy, and if they knew anything about LAM, were collected at timepoints T0 and T1. Questions specific to the duration of exclusive breastfeeding were asked at timepoint T3. The potential predictors considered were the two study arms (GANC vs. IANC), time (T0 vs. T1 or T3 as relevant), the mother’s education, and whether this was their first pregnancy. Tracking logs were used to track participants throughout the study. Incentives included baby items (socks, hand gloves, and dresses) for participants at each data collection time point.

### Data analysis

Data were analyzed using summary statistics based on mean, SD, or frequency to characterize the sample distribution of each arm. Categorical data were compared using the chi-square test. Maternal age and wealth index were tested using a t-test, while the number of previous pregnancies was assessed using the Mann-Whitney Wilcoxon test. For categorical items, the results are presented as percentages, whereas for continuous items, the means and standard deviations are reported, with a p-value of less than 0.05 denoting significance. Questions assessed participants’ awareness and knowledge of LAM. The responses to the outcomes considered were all dichotomous in nature. At T1, about 13% of the data was missing, and about 30% at T3, with data missing at random (MAR). Multiple imputations with 100 iterations were done. Mixed-effect logistic regression models were applied to the imputed data to test the effect of the explanatory variables on the different outcomes. A pooled Wald F-test was used to evaluate the overall significance of the model predictors. The effect of the intervention over time (i.e., test for the difference of differences) is the test of simple interaction between time and study group in the mixed effects logistic model, where the p-values and the corresponding 95% CI are derived from a pooled t-statistic, pooled from the multiply imputed datasets using Rubin’s rule. The covariates considered were maternal education and parity. The results section below summarizes additional details about these outcome measures and explanatory variables.

## Results

The overall number of participants enrolled in the study was 1761. The majority of the participants (56%) were in the age group of 25–34. Twenty-eight percent (28%) were less than 25 years of age, and 16% were 35 years or older. The majority, 96%, were either married, cohabiting, or living with their partners. For education, 63% had junior high school education or less, 27% had secondary education, and 10% had a tertiary education. Twenty percent (20%) of the women were pregnant for the first time, and 97% had a hospital, polyclinic, or health center identified as their location for delivery (See Table 1). There were no significant differences between participants in the intervention and control groups for any of the demographics considered.

**Table 1:** Characteristics of participants enrolled.

Categorical Variables n (%)		Control (I-ANC)	Intervention (G-ANC)	P-value
		N = 884	N = 877	
Age Category				
	Less than 25	266 (30%)	235 (27%)	0.193
	25-34	477 (54%)	510 (58%)	
	35 or more	141 (16%)	132 (15%)	
Maternal Education				
	Primary	120 (14%)	126 (15%)	0.6895
	Middle/JHS/JSS	429 (50%)	400 (47%)	
	Secondary/SHS/Technical/Vocational	223 (26%)	236 (28%)	
	Tertiary	83 (10%)	81 (10%)	
Partner Education				
	Middle/JHS/JSS or less	335 (40%)	331 (38.5%)	0.8525
	Secondary	306 (37%)	321 (37%)	
	Tertiary	126 (15%)	135 (16%)	
	N/A, Unknown	64 (8%)	73 (8.5%)	
Religion				
	Christianity	835 (95%)	811 (92%)	0.1185
	Muslim	39 (4%)	58 (7%)	
	Other	10 (1%)	8 (1%)	
First pregnancy				
	No	703 (80%)	709 (81%)	0.4876
	Yes	181 (20%)	168 (19%)	
Location of Delivery				
	Hospital/Polyclinic/Health Center	853 (96%)	858 (98%)	0.0904
	Other	31 (4%)	19 (2%)	
**Continuous Items: Mean (SD)**				
Maternal age		28.1 (6)	28.3 (5.6)	0.5042
Wealth index		6.9 (2.4)	6.9 (2.3)	0.6174
Number of previous pregnancies		3.5 (1.4)	3.5 (1.5)	0.7075

Table 2 presents the results of the assessment of knowledge of breastfeeding and LAM among participants. Odds ratios (ORs) along with 95% confidence intervals (95% CI) and p-values from the mixed-effect logistic models are summarized in the table. For the question, “Have you heard about breastfeeding for pregnancy prevention?”, the results from the mixed effects logistic model show the overall model was statistically significant: F(7, 24567.8) = 43.63, p < 0.001, indicating that the predictors jointly contributed to the outcome. The OR for the time and group interaction effect is 3.9 (95% CI: 2.7, 5.7, p < 0.001), indicating that the odds of having heard about breastfeeding for pregnancy prevention were nearly fourfold in the GANC as compared to IANC from T0 to T1. In addition, education and first pregnancy status were also found to be significant predictors. The odds of having heard about breastfeeding to prevent pregnancy are significantly higher (between 4–6 times, 95% CI = 2.5–10) for tertiary-educated women compared to women with less education. No significant differences were observed between women with primary, middle, and secondary education. The odds of having heard about breastfeeding to prevent pregnancy were 60% lower (OR=0.4, 95% CI: 0.3, 0.5, p < 0.001) for women who were pregnant for the first time compared to women who had been pregnant before.

**Table 2:** Knowledge of LAM among participants.

1. Have you heard about breastfeeding for pregnancy prevention?				
		Odds ratio	P-value	95% Confidence interval
Study arm	IANC	(ref)	0.874	
	GANC	0.98		0.8, 1.2
Time	T0	(ref)	< 0.001	
	T1	2.7		2.1, 3.5
**Interaction: Study arm and Time**	(reference)		< 0.001	
	Study arm = GANC, Time = T1	3.9		2.7, 5.7
**First pregnancy**	No	(ref)	<0.001	
	Yes	0.4		0.3, 0.5
**Education**	Tertiary	(ref)		
	Primary	0.17	< 0.001	0.2, 0.3
	Middle	0.21	< 0.001	0.1, 0.3
	Secondary	0.24	< 0.001	0.2, 0.4
**2. What do you know about LAM: Nothing**				
Study arm	IANC	(ref)	0.081	
	GANC	0.8		0.7, 1.0
Time	T0	(ref)	< 0.001	
	T1	0.6		0.5, 0.7
**Interaction: Study arm and Time**	(reference)		< 0.001	
	Study arm = GANC, Time = T1	0.3		0.2, 0.5
First pregnancy	No	(ref)	<0.001	
	Yes	2.8		2.1, 3.6
Education	Tertiary	(ref)		
	Primary	4.7	< 0.001	3, 7.1
	Middle	3	< 0.001	2.1, 4.3
	Secondary	2.5	< 0.001	1.7, 3.6

In response to the question, “What do you know about Lactational Amenorrhea as a method to prevent pregnancy: nothing?” recorded at enrollment (T0) and again at T1 (34 weeks of gestation – 3 weeks post-delivery), the mixed-effects logistic model results showed overall statistical significance: F(7,25585.8) = 35.57, p < 0.001, indicating that the predictors jointly contributed to the outcome. The odds ratio (OR) for the time and group interaction effect is 0.3 (95% CI: 0.2, 0.5, p < 0.001), indicating that the odds of having heard nothing about LAM decreased more than threefold in the GANC as compared to IANC from T0 to T1. Compared to individuals with tertiary education, the odds of knowing nothing about LAM are 2.5–4.7 times higher for secondary, middle, or primary educated women. Primary educated women had higher odds of knowing nothing about LAM compared to middle and secondary educated women, indicating that lower education is associated with significantly higher odds of lacking education about LAM. Finally, those who were pregnant for the first time had higher odds (OR=2.8, 95% CI = 2.1, 3.6) of knowing nothing compared to women who were pregnant before.

Marginal predicted probabilities are presented in Table 3 to illustrate the differences in knowledge of breastfeeding as a method of pregnancy prevention and awareness of LAM across time, study arms, education levels, and pregnancy history. These probabilities provide an intuitive understanding of group differences, complementing the odds ratios.

**Table 3:** Expected Probabilities on Knowledge of LAM among Participants.

1. Have you heard about breastfeeding for pregnancy prevention?		
Study arm and time	Predicted probabilities (%)	95% CI
Time = 0		
IANC	33.4	29.6, 37.5
GANC	33.0	29.2, 37.1
Time = 1		
IANC	57.8	53.1, 62.4
GANC	84.2	79.9, 87.4
**Education**		
Primary	44.3	37.8, 51.1
Middle	49.9	46.3, 53.5
Secondary	52.8	47.8, 57.6
Tertiary	82.5	76.5, 87.2
**First pregnancy**		
Not a first pregnancy	58.5	55.6, 61.4
First pregnancy	33.9	28.9, 39.4
**2. What do you know about LAM: Nothing?**		
Time = 0		
IANC	61.4	57.4, 65.2
GANC	56.5	52.5, 60.4
Time = 1		
IANC	48.5	44.0, 52.9
GANC	18.5	15.0, 22.5
**Education**		
Primary	57.8	51.6, 63.7
Middle	47.4	43.9, 50.9
Secondary	42.3	37.8, 47.0
Tertiary	22.9	17.5, 29.4
**First pregnancy**		
Not a first pregnancy	40.0	37.3, 42.7
First pregnancy	64.7	59.4, 69.7

At T3 (6 months postpartum), participants were also asked how old the baby was when they began giving other liquids. A logistic regression on the multiple imputed data was done to compare the two groups. The odds of feeding their baby other liquids before 6 months were significantly lower in the GANC (OR=0.61, 95% CI: 0.48, 0.77, p < 0.001) compared to IANC. Women in the GANC group had 39% lower odds of feeding other liquids to their baby before 6 months compared to the IANC group. Lastly, the participants were asked about the family planning method currently being used. Only 8 (1.2%) chose LAM in IANC, and 18 (2.9%) chose LAM in GANC.

## Discussion

This study examined the impact of G-ANC on women’s awareness and knowledge of LAM. The findings showed that participation in G-ANC was strongly associated with greater awareness of breastfeeding as a method of pregnancy prevention, as well as improved knowledge of LAM over time. This result suggests that GANC has the potential to effectively promote maternal and child health outcomes. Awareness and knowledge are vital for enabling behavior change [42–44]. The group model provides a supportive and interactive environment and incorporates targeted family planning education into antenatal group sessions. Through peer-to-peer learning and professional counseling, G-ANC effectively delivers family planning information, especially for LAM. This reinforces existing evidence that group care fosters comprehensive counseling, facilitates discussions during G-ANC sessions, and provides repeated exposure to key messages, which leads to awareness, the retention of health information, empowerment, adherence, and the application of family planning knowledge [10,11,30,45–48].

The results also indicate that parity and education are strong predictors of LAM awareness and knowledge. Research shows that multiparous women and mothers with higher educational backgrounds typically tend to have greater awareness and understanding of LAM than women with less education [49–51]. Studies conducted by Ambike et al. and Wakuma et al. [52,53] in India and Ethiopia highlight that first-time mothers are typically inexperienced and lack prior knowledge about LAM. This has been found to be mostly pronounced among adolescents who are typically vulnerable, have less maternal experience, and often struggle with limited knowledge due to lower educational attainment at their age [12,13]. The observed differences may be attributed to varying levels of experience [54], access to information, and the ability to make informed choices [17,53]. Additionally, varying factors such as distance from health facilities, fertility desire, place of delivery, convenience, support, and counseling received about LAM are all associated with knowledge and use of the method [49,50,55]. These determinants highlight the persistent role of equity in shaping health outcomes, which is heavily considered in the G-ANC approach to care, aligning with Sustainable Development Goal 3.7, which seeks to ensure universal access to sexual and reproductive healthcare services, including family planning, information, and education [56]. This strategy is crucial for ensuring that no one is left behind in accessing reproductive health services [10,57].

Taken together, these findings highlight the promise of G-ANC as an innovative platform to contextually tailor and advance postpartum family planning in low-resource settings where modern methods are often limited. By embedding structured counseling on LAM within group sessions, programs can simultaneously address knowledge gaps, strengthen maternal self-efficacy, and promote informed contraceptive choices. Future research should examine strategies to extend support into the postnatal period, ensuring that gains in awareness and knowledge translate into sustained correct practice and timely transition to other modern contraceptive methods once LAM criteria are no longer met. Additionally, evaluating adolescent-specific adaptations and equity-focused interventions will be essential to ensure that G-ANC benefits women across parity, educational, and socio-economic gradients.

## Strengths and limitations

Participants in this study were limited to the Eastern Region of Ghana, and therefore, our findings may not apply to a larger population of women. The study’s reliance on self-reported data on breastfeeding and contraceptive practices might also introduce bias or inaccuracies; however, valid instruments were used to mitigate this limitation. This study also has multiple strengths. A rigorous methodological design (cRCT) was used to evaluate the effect of GANC on LAM knowledge and uptake with a large sample size. Additionally, the study is strengthened by participant follow-up across the different time points to examine change over time and differences between groups.

## Conclusion

The study is the first we are aware of to investigate the impact of GANC as an antenatal care intervention to improve awareness and knowledge of LAM in Ghana. LAM is an inexpensive and effective family planning method crucial for improving the health of mothers and babies. Creating awareness and increasing knowledge through group care is an effective technique for influencing the usage of this method. Leveraging on the concept of knowing, which is a fundamental component of GANC towards improving behavior change, can be used to bridge the knowledge gap and all other limitations highlighted by this study’s findings.

## Supporting information

S1 Checklist S1 Consort Checklist. (DOC)

## Abbreviations

FGD: Focused group discussion
GANC: Group antenatal care
LAM: Lactational amenorrhea method
RA: Research assistants
SDG: Sustainable Development Goal
UN: United Nations
WHO: World Health Organization
